# Barriers and Facilitators to Older Adults’ Acceptance of Camera-Based Active and Assisted Living Technologies: A Scoping Review

**DOI:** 10.1093/geroni/igae100

**Published:** 2024-11-29

**Authors:** Natalie An Qi Tham, Anne-Marie Brady, Martina Ziefle, John Dinsmore

**Affiliations:** Trinity Centre for Practice and Healthcare Innovation, School of Nursing and Midwifery, Trinity College Dublin, Dublin, Ireland; Trinity Centre for Practice and Healthcare Innovation, School of Nursing and Midwifery, Trinity College Dublin, Dublin, Ireland; Chair of Communication Science, Human-Computer Interaction Center, RWTH Aachen University, Aachen, Germany; Trinity Centre for Practice and Healthcare Innovation, School of Nursing and Midwifery, Trinity College Dublin, Dublin, Ireland

**Keywords:** Active and assisted living, Ambient assisted living, Behavior change, Computer vision, Technology acceptance

## Abstract

**Background and Objectives:**

Camera-based active and assisted living (AAL) technologies are an eminent solution to population aging but are frequently rejected by older adults. The factors that influence older adults’ acceptance of these technologies remain poorly understood, which may account for their lagging diffusion. This scoping review aimed to identify the barriers and facilitators to older adults’ acceptance of camera-based AAL technologies, with a view to facilitating their development and widespread dissemination.

**Research Design and Methods:**

MEDLINE, CINAHL, Embase, IEEE Xplore Digital Library, ACM Digital Library, Web of Science, and gray literature databases were searched from inception to June 2024. Publications that reported data on barriers and facilitators to the acceptance of camera-based AAL technologies among community-dwelling older adults aged 60 and above were eligible. Barriers and facilitators were extracted and mapped to the theoretical domains framework, thematically clustered, and narratively summarized.

**Results:**

A total of 28 barriers and 19 facilitators were identified across 50 included studies. Dominant barriers concerned the technology’s privacy-invasive, obtrusive, and stigmatizing qualities. Salient facilitators included the perceived usefulness of, and older adults’ perceived need for, the technology.

**Discussion and Implications:**

Results inform practitioners’ selection of strategies to promote older adults’ acceptance of camera-based AAL technologies. These efforts should transcend the conventional focus on pragmatics and give credence to psychological, social, and environmental influences on technology acceptance.

**Translational Significance:** Camera-based active and assisted living (AAL) technologies have the potential to optimize lifespan and mitigate the burdens of population aging but are slow to be incorporated into older adults’ homes. To date, the factors that influence older adults’ acceptance of these technologies are understudied, which may account for the technology’s lagging diffusion. By uncovering the barriers and facilitators to said acceptance and delineating how these span psychological, social, and environmental domains, the results of this scoping review stand to inform the development and dissemination of more acceptable—and thus more widely used—camera-based AAL technologies.

Global nations are facing a crisis in demography that, if insufficiently addressed, poses a significant risk to the welfare of societies. Projections estimate a twofold increase in the number of individuals aged 60 and above between 2015 and 2050, to make up almost 22% of the global population (1). If current trajectories prevail, older adults could represent one in every 5 persons by 2050, up from 1 in 8 in 2015 (1). Population aging poses severe societal implications. Chronic diseases accumulate with age and, as a leading cause of morbidity and mortality, place immense upward pressures on health and long-term care systems (2). In response to these socioeconomic threats, policy discourses have given renewed voice to an “aging-in-place” agenda, which aims to decentralize the provision of costly acute and long-term care to the home environment by enabling older adults to remain in their own homes for as long as possible (3). This is envisaged to alleviate the strain on healthcare systems while fulfilling older adults’ desires to remain at home rather than in institutional care as they age (3).

Active and assisted living (AAL) technologies—broadly understood as the use of ubiquitous information and communication technologies to facilitate secure, better-quality lives for older adults—have been conceived as prime enablers of aging in place (4). In particular, camera-based AAL technologies offer a means for state-of-the-art care and support for older persons (5). By leveraging techniques in computer vision and artificial intelligence, camera-based AAL technologies can detect falls and signal for emergency assistance where necessary (6), automate safer and more comfortable living environments (7), and even administer preventive interventions to target morbidity upstream (8). In one instructive example, Andreu et al. (9) employed depth cameras to extract from users’ facial features various indicators of cardiometabolic risk such as overweight and obesity. Indicators were then coalesced into individual risk profiles against which tailored recommendations for disease self-management were made, such as suggestions to modify one’s diet or physical activity. With promise, the researchers noted 3-month reductions in individuals’ cardiometabolic risk following the use of the technology (9).

Despite their merit, camera-based AAL technologies are slow to be incorporated into people’s homes (10). This lagging diffusion has typically been rationalized with reference to utilitarian considerations à la traditional models of technology acceptance. As an empirical mainstay, the Technology Acceptance Model (TAM) posits that individuals’ acceptance of a particular technology can be attributed to its perceived usefulness and ease of use (11). Indeed, pragmatic barriers to older adults’ acceptance of camera-based AAL technologies are well documented. These include, for instance, the need to specially retrofit the home for unoccluded monitoring, which can prove an elaborate and costly affair (12), as well as limited digital literacy among older adults (13).

Despite its predictive utility, the TAM has been critiqued on account of its neglect of other antecedents to technology acceptance. Recent views suggest that failed AAL technology deployments have less to do with instrumental issues than with the problematic stereotypes conveyed by these technologies. To Neven and Peine (14), AAL technologies frame old age as a period of dependency and deterioration and are therefore imbued with a stigmatizing quality that stymies their acceptability. Similarly, Lupton and Seymour (15) concur that it is the jarring discrepancy between older adults’ desired identities—for example, as self-reliant and vital—and the stereotyped identities articulated by AAL technologies—for example, as dependent and decrepit—that motivates rejection. This is evident, for example, in how AAL technologies are often dismissed by older adults as only benefiting the “crippled” (16), “invalid” (17), or “very handicapped” (18)—social categories to which they denounce belongingness.

Arguably the chief source of antipathy toward camera-based AAL technologies is their intrusive quality. Even where older adults concede to using AAL technologies, this willingness often precludes the use of cameras (19–21). Berridge (22) attributes this resistance to the ways in which cameras sever the home from cherished connotations of refuge and cocooning and instead imbue it with a sense of disconcerting exposure and vulnerability. Prevalent in the literature are allusions to aversive notions of being “watched” (13,23) or “spied on” (13) by “Big Brother” (23,24) that disenchant otherwise inclined users. One study found that when subject to camera-based monitoring, older adults engaged in deliberately sabotaging behaviors such as obstructing camera lenses, hiding behind furniture, and walking backward to avoid facial detection, which ultimately diminished the technology’s effectiveness (25).

Importantly, older adults’ resistance to camera-based AAL technologies is not unyielding. Crucial observations have been made about trade-offs that occur in older adults’ cognitive assessments of such technology, where the risks of adoption are weighed against the accordant benefits (26). For instance, older adults are inclined to sacrifice notions of privacy and dignity for comparatively weightier benefits such as improved health, safety, and independence (27). Additionally, older adults tend to relinquish their privacy to camera-based monitoring if they perceive a genuine need for care, or if doing so is seen as the sole recourse to avoiding residential care (21,28).

Overall, there exists a panoply of factors that determine whether camera-based AAL technologies are appropriate or rejected. Although the factors that influence the acceptance of non-visual AAL technologies are well-chronicled (29), those that influence the acceptance of camera-based AAL technologies are understudied. Furthermore, where camera-specific determinants have been scrutinized, studies have focused narrowly on pragmatic issues to the neglect of other important psychological, social, and environmental factors. This fragmented theorizing may explain older adults’ limited acceptance and adoption of camera-based AAL technologies.

## Conceptual framework

To increase people’s acceptance and adoption of new technologies is to change their behavior. There is empirical consensus that attempts to change behavior benefit from the application of theory (30). Theory provides a useful framework against which the determinants of behavior and corresponding behavior change techniques can be identified and has recognized utility for informing the development of behavior change interventions (30). For instance, theory can facilitate the identification of theoretical constructs to target in an intervention (eg, beliefs about capabilities), individuals to target within an intervention (eg, individuals with poor beliefs about their capabilities), and specific behavior change techniques (eg, verbal encouragement to increase individuals’ beliefs about their capabilities) that might be used in an intervention to elicit the desired behavior (31).

## The Theoretical Domains Framework

The Theoretical Domains Framework (TDF) provides a useful theoretical basis against which behavioral determinants might be conceptualized. The TDF outlines 14 theoretical domains for understanding and changing behavior: (i) *knowledge*; (ii) *skills*; (iii) *social/professional role and identity*; (iv) *beliefs about capabilities*; (v) *optimism*; (vi) *beliefs about consequences*; (vii) *reinforcement*; (viii) *intention*; (ix) *goals*; (x) *memory, attention, and decision processes*; (xi) *environmental context and resources*; (xii) *social influences*; (xiii) *emotion*; and (xiv) *behavioral regulation* (32). By informing the underlying philosophy and content of behavior change interventions, the TDF has benefitted the understanding of the drivers and impediments to older adults’ behavior, including in such domains as physical activity (33) and medication adherence (34).

A TDF-based analysis of the barriers and facilitators to older adults’ acceptance of camera-based AAL technologies is pertinent for 2 main reasons. First, delineating the barriers and facilitators that pertain to the technology’s form and function will inform the a priori design of more acceptable camera-based AAL technologies. Second, delineating the range of factors influencing acceptance can inform the development of behavior change interventions aimed at improving older adults’ acceptance and use of said technology.

To the best of our knowledge, no prior study has attempted a theoretical analysis of said acceptance. This scoping review therefore aimed to identify, with categorization to the TDF, the barriers and facilitators to older adults’ acceptance of camera-based AAL technologies.

## Methods

### Protocol

This review has been conducted in accordance with established scoping review methodology from the Joanna Briggs Institute with guidance from a prepublished study protocol (35). As the literature on the acceptance of camera-based AAL technologies is embryonic and methodologically heterogenous, a scoping review was appropriate given its capacity to synthesize evidence in emerging and fragmented knowledge landscapes (36). The protocol follows the Preferred Reporting Items of Systematic Reviews and Meta-Analysis for Scoping Reviews (PRISMA-ScR) methodology to ensure transparent, systematic, and unbiased conduct of the review (37).

### Eligibility Criteria

Studies were assessed for eligibility against a Population, Concept, and Context framework:

### Population

Studies were eligible if they included community-dwelling older adults aged ≥60 years as participants, or if they included a subgroup analysis of community-dwelling older adults with a mean age ≥60 years. This review adopts the United Nations’s definition of an “older adult” as any individual aged 60 and over (1), driven by the notion that individuals can benefit from using AAL technologies even before the onset of statutory pension eligibility age (38). Indeed, research has demonstrated good efficacy of AAL technologies for older adults aged ≥60 years in such domains as chronic disease self-management (39) and fall detection (40), and, importantly to this study, studies investigating the determinants of AAL technology acceptance have sampled from populations aged 60 and above (41).

Studies that included other stakeholder groups in addition to older adults (eg, family members and informal caregivers) were excluded unless data specific to older adults could be separately extracted.

### Concept

Articles were considered for inclusion if they discussed camera-based AAL technologies, defined as technology that incorporates the use of cameras in individuals’ daily living environments to enable them to “stay active longer, remain socially connected, and live independently into old age” (4). A defining feature of such technology is a central processing component that receives and analyses camera streams such as images or videos (5). Studies were therefore excluded if the technology under evaluation did not feature a central processing point, such as in telehealth applications where cameras simply facilitated real-time communication between patient and physician.

Studies were excluded if they investigated wearable cameras—that is, cameras attached to the user’s body—rather than environmental cameras—that is, cameras mounted on walls, ceilings, or the like (42). This decision was appropriate given the differential privacy impacts of wearable versus environmental cameras (43). Environmental cameras monitor individuals from a third person point of view and are therefore amenable to sensitive features such as facial data. Such sensitive data typically eludes wearable cameras that monitor the environment from the user’s point of view (43). Thus, for pragmatic and evaluative parsimony, studies focused on wearable cameras were excluded.

Eligible studies also needed to report on barriers and facilitators to acceptance, defined as any factor, characteristic, view, or belief that impedes or enables older adults’ acceptance of camera-based AAL technologies (44). Studies that used proxy measures of acceptance (eg, adoption or intention to adopt the technology) were eligible.

### Context

Studies needed to sample from private residential settings such as private homes or homes in retirement communities. This decision was premised on the notion that AAL technologies are intended to support individuals in their natural living environment (ie, the home) and to circumvent transitions to residential care (3). Accordingly, studies that sampled from non-private settings such as residential care or assisted living facilities were excluded.

### Identifying Relevant Studies

We considered academic articles of any study design (quantitative, qualitative, and mixed-methods) that reported primary data. Secondary and non-empirical sources such as reviews or opinion pieces were excluded to avoid including data from single or limited perspectives. However, reference lists of secondary sources were screened for potentially relevant original articles.

Eligible articles were identified across 6 key databases: Medline, CINAHL, Embase, Web of Science, ACM Digital Library, and IEEE Xplore Digital Library. Preliminary database searches revealed a scarcity of studies that contained, at least in the title and abstract search fields, terms related to camera-based AAL technologies (eg, “cameras,” “video,” and “visual”). For similar reasons and following prior studies (45), it was anticipated that terms relating to “barriers” and “facilitators” would only be discussed in the full texts. Therefore, to avoid prematurely excluding relevant literature, search strategies excluded references to both camera-based technology and barriers and facilitators.

Database-specific search strategies were developed with guidance from an expert subject librarian to probe 4 main concepts: (i) active and assisted living; (ii) older adults; (iii) the home setting; and (iv) acceptance. An example MEDLINE search strategy is presented in Supplementary Table 1 in Supplementary Material.

To ensure coverage of the breadth of available material, grey literature searches were conducted on Google Scholar. Search queries featured the following terms: “older adult*” (Population), “assist* living” (Concept), “ambient assist*”(Concept), “accept*” (Concept), “home*” (Context). Title-only screenings were conducted across the first 200 returned search records to ensure that no relevant publications were missed.

The initial database search was conducted in September 2021. Updated searches were conducted in May 2022, March 2023, and June 2024 to capture any eligible studies published in the intervening period between searches. No date or language restrictions were applied. The reference lists of identified articles were hand-searched for additional citations, and where necessary, the authors of papers were contacted to obtain full texts.

### Study Selection

All search results were collated and imported into EndNote 20.1 for de-duplication. The remaining citations were imported into Covidence systematic review management software for screening. Titles and abstracts were assessed against the predefined eligibility criteria by 2 independent reviewers and obviously irrelevant studies were excluded. Questionable records underwent inter-reviewer discussion and decisions on eligibility were rendered jointly. Studies with ambiguous eligibility were retained and full texts were reviewed independently and in duplicate. At this stage, particular focus was placed on ascertaining the presence of camera components in the AAL technology under study, as well as any reference to barriers and facilitators. Where consensus on an article’s eligibility could not be achieved, a third reviewer with expertise in digital health and behavior change research was consulted for arbitration to yield a final result set.

### Data Extraction

A prepiloted data extraction form was used to tabulate information on study classifiers (eg, authors and publication year), study characteristics (eg, study aim, design, and participants), information on the AAL technology under evaluation (eg, type of camera used and context of usage), and study outcomes (eg, barriers and facilitators). The extraction process was independently piloted on an initial subset of studies by 2 reviewers to ensure parsimony in extraction and completeness of the extracted data. Subsequently, the lead reviewer executed data extraction across all identified studies.

Given the heterogeneity of acceptance-related outcomes that were evaluated in the included studies (eg, qualitative, quantitative, or both), findings were synthesized using a convergent integrated approach (46). This involved transforming quantitative data into textual descriptions that were then pooled with and examined alongside qualitative data extracted verbatim (46). Narrative synthesis was used to generate integrated findings based on the pooled data in ways that answered the review questions (46).

### Data Analysis

Next, barriers and facilitators were categorized to the TDF domains using directed content analysis (47) with guidance from a prespecified data coding manual (Supplementary Table 2). Using a deductive approach, selections of text were coded to represent barriers and facilitators, which were then mapped onto the 14 TDF domains. As TDF domains are not mutually exclusive and important interrelations exist between domains (48), barriers and facilitators were coded to more than one domain if deemed appropriate. The coding process was independently piloted by 2 researchers on a subset of studies. Discrepant codes were resolved through inter-reviewer discussion with third-reviewer arbitration where necessary. Coding was thereafter undertaken across all studies by the lead reviewer.

An inductive, data-driven analysis was thereafter undertaken to delineate more defined themes within each TDF domain. Specifically, codes within each TDF domain were thematically analyzed and interrelated codes were then clustered into themes (49). For example, barriers identified in the *beliefs about consequences* domain relating to the intrusive and burdensome nature of camera-based AAL technologies were coded as separate “Privacy and data protection concerns” and “Technical issues and burdens” themes. Themes were inductively derived from the data by the lead reviewer and underwent review by the expert third reviewer to ensure that they were appropriately coded and sufficiently distinct.

## Results

**Figure 1.**
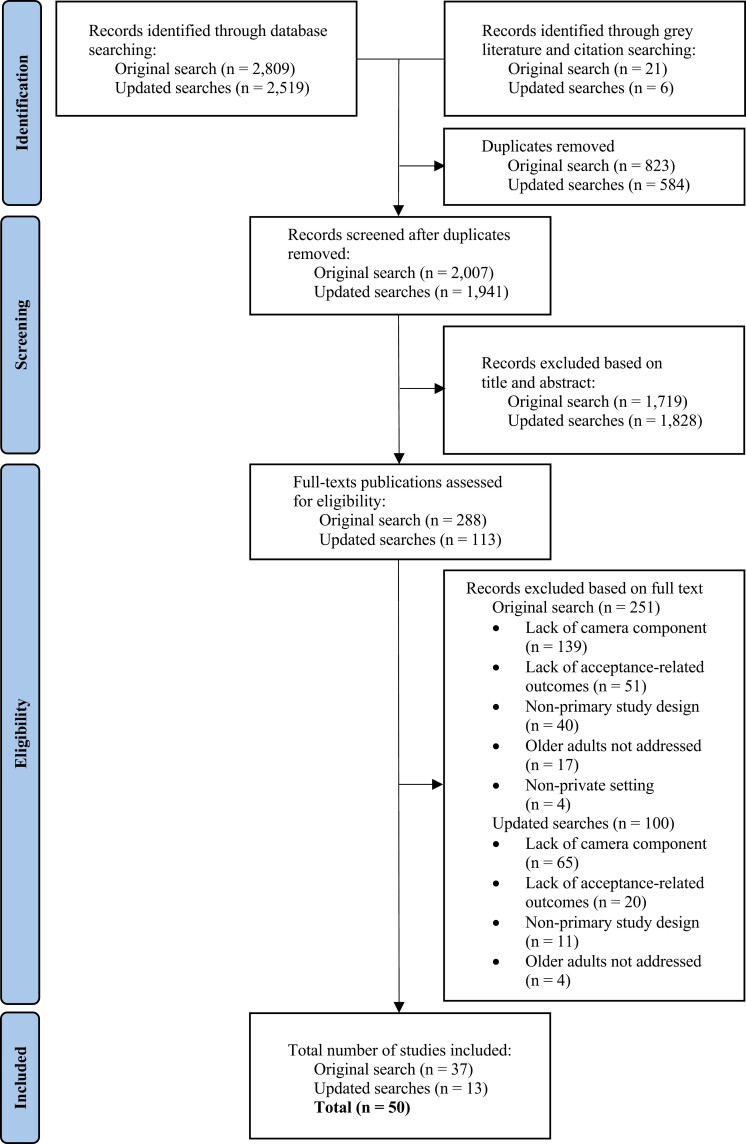
PRISMA flowchart of study selection process.

The original search yielded a total of 2 007 nonduplicate records, with 1 941 additional records retrieved during updated searches. Irrelevant articles were removed following title and abstract screening, yielding 401 potentially relevant publications. Of these, 351 failed to meet the inclusion criteria, resulting in a final analytic pool of 50 articles. Thus, of the total number of nonduplicate articles retrieved, only a minority (1.27%) addressed the factors influencing older adults’ acceptance of camera-based AAL technologies. Figure 1 illustrates progress through the review.

### Study Characteristics

Characteristics of the included studies are summarized in Supplementary Table 3. The included studies were published between 2003 and 2023, though the majority (94%) were published in or after 2007. This post-2007 upsurge in research on AAL technologies corresponds with the official launch of the Ambient Assisted Living Joint Programme by the European Commission (50). Most studies were of qualitative design (56%), with a proportionately smaller number of quantitative (26%) and mixed-method (18%) studies. All but 4 (51–54) studies were conducted in Western countries, and all but one (55) was available in English.

Most studies (68%) sampled exclusively from older adult populations, though some included combined samples of older adults and their family members (8%), healthcare professionals (6%), formal and/or informal caregivers (4%), or some combination of the above (4%). Some (10%) studies considered the general adult population but were included as data specific to older adults could be separately extracted. Population samples of older adults ranged from *n* = 5 to *n* = 756, with mean ages (where reported) ranging from 62.3 years to 86.9 years. Of the studies that reported information on participants’ gender, an overwhelming proportion (82.5%) were over-represented by females.

### Types of Camera-Based AAL Technologies

The majority of studies included standard Red-Blue-Green (RGB) cameras as their only visual component (19–21,23,24,28,41,51–54,56–75). Where alternative modalities of visual sensing were evaluated, these featured the use of depth cameras (55,66), thermal cameras (76,77), combinations of different types of cameras (such as in the Microsoft Kinect, which incorporates both RGB and depth cameras) (13,78–81), and camera-equipped social robots (82–87). Twelve studies evaluated camera-based AAL technologies alongside the use of privacy filters (27,55,66,76,78–81,84,88–90)—that is, image processing techniques that redact or obscure potentially identifying data such as facial detail (91). Here, monitored individuals are effectively anonymized, as only their “shape” (79), silhouette” (27,88–90), or “outline” (78) can be discerned. The types of camera-based AAL technologies evaluated in the included studies are summarized in Supplementary Table 4.

### Barriers and Facilitators and Relevant Theoretical Domains

A total of 28 barriers and 19 facilitators were identified across 12 TDF domains: *knowledge, skills, social/professional role and identity, beliefs about capabilities, optimism, beliefs about consequences, reinforcement, goals, memory, attention, and decision processes, environmental context and resources, social influences,* and *emotion*.

Table 1 summarizes key barrier and facilitator subthemes identified across the included studies, with categorization by TDF domain and presentation in order of importance. Coding frequency (ie, the total number of times a barrier/facilitator was coded, expressed as absolute numbers and as a percentage of the total number of identified codes) was used as a proxy for importance. Facilitators were occasionally reported as the opposite of a barrier and vice versa.

**Table 1.** Barriers and Facilitators to Older Adults’ Acceptance of Camera-Based AAL Technologies Categorized by TDF Domain, Presented in Order of Importance.

TDF domain	Coding frequency ^a^	Barriers	Facilitators
Beliefs about consequences	336, 33.8%	Privacy and data protection concerns	Perceived usefulness
		Technological issues and burdens	Assurance of data security
		Perceived lack of usefulness	Anonymization of camera-acquired images
		Concern about potential to create unnecessary worries	Perceived capacity for the system to facilitate social relationships
			Perceived increase in privacy through usage of the technology
Social/Professional Role and Identity	145, 14.6%	Concern about loss of autonomy and/or dignity	Perceived need for the technology
		Perceived lack of need for the technology	
		Perceived stigma	
		Self-other distinction in perceived need for the technology	
		Assimilation of “old-fashioned” or “technophobic” identity	
Environmental Context and Resources	144, 14.5%	Costliness	Unobtrusiveness
		Obtrusiveness	Ease of use
		Low ease of use	
		Lack of integration with existing lifestyle	
		Lack of accommodating home infrastructure	
		Presence of existing AAL technology at home	
Social Influences	88, 8.86%	Preference for human-provided care and interaction	Social and/or technical support for using the technology
		Concern about caregiver burden	
		Presence of existing care and/or support network	Perceived potential to relieve caregiver burdens
			Normative signals for uptake of the technology
			Social robot seen as companion
Reinforcement	83, 8.36%	Lack of prior experience with (assistive) technology	Prior experience with (assistive) technology
		Lack of prior health adversity	Prior health adversity
			Prior receipt of care at home
Beliefs about capabilities	53, 5.34%	Perceived lack of self-efficacy for using the technology	Perceived control over the technology
Memory, attention, and decision processes	47, 4.73%	Perceived lack of current (versus future) need for the technology	Willingness to trade privacy for improved health, safety, and independence
Emotions	45, 4.53%	Discomfort around cameras	Fear of being helpless during emergencies
		Technology anxiety	
		Embarrassment and/or shame	
Knowledge	23, 2.32%	Lack of knowledge and/or understanding of the technology	
Goals	13, 1.31%		Desire to avoid transition to residential long-term care
Optimism	9, 0.91%	Unrealistic optimism about own health and aging	
Skills	7, 0.70%	Lack of skills for competent usage of the technology	

*Note*. AAL = active and assisted living; TDF = Theoretical Domains Framework.

^a^The number of times the theme was coded, expressed in absolute numbers and as percentage of the total number of identified codes).

The section below provides a narrative description of barriers and facilitators with categorization according to the TDF and in order of importance.

### Barriers and Facilitators With Critical Importance

The TDF domains with the greatest representation across all included articles, being represented in over 60% of all identified codes, were: *beliefs about consequences* (coding frequency = 33.8%), *social/professional role and identity* (14.6%), and *environmental context and resources* (14.5%).

### Beliefs About Consequences

Privacy and data protection concerns emerged as the primary barrier to older adults’ acceptance of camera-based AAL technologies (13,19–21,24,28,51–54,56,59–78,82,83,85,88–90). Testifying to the particular aversiveness of cameras, numerous studies found that although older adults conceded to their homes being retrofitted with non-visual AAL technologies such as motion sensors or microphones, the use of cameras was unanimously rejected on account of their intrusive quality (23,24,65,73). Acceptance also dwindled where older adults were concerned about the security of their personal data (13,20,24,28,51–53,56,65,67,69,72,75,76,83,85,88,89), with many voicing concern about their potential exposure to malicious third-party activity (13,20,56,65,89). As noted by one older adult: “People get their identity stolen! […] People are worried about their facial data” (56).

 Camera-based AAL technologies had the potential to create unnecessary burdens on account of technical failures, such as by triggering false alarms during non-emergency situations, which was another notable barrier to acceptance (13,20,63,67,68,70,74,76,81,84,89,90). Acceptance was also diminished among older adults who were unconvinced about the technology’s benefits, such as its capacity to reliably summon help during emergencies (20,51,67,70,89), to intervene in the seemingly immutable aging trajectory (19,71,72,82), or to prevent the transition to residential care (71,82).

Nonetheless, older adults’ privacy concerns were contextually sensitive, being diminished where cameras were considered in less sensitive spaces such as living rooms or kitchens, and heightened in more intimate spaces such as bathrooms or bedrooms (13,23,66,71,77,78). One provocative finding was that some older adults believed that a camera-surveilled home had *enhanced* rather than diminished privacy (23,41,74). To these older adults, environments such as residential care had forgone privacy due to the frequent influx of other “old dodderers” (23). A technologically retrofitted home was seen to circumvent these intrusions and thus was considered comparatively more private and acceptable (23,41,74).

Additionally, camera-based AAL technologies were better received to the extent that they were seen as useful (13,19,20,24,41,51–53,55,59,62–65,67–69,71,72,74,76,78,79,81–84,86–90). It was important to older adults that they would receive timely help “if something were to happen” (79). The features that distinguished camera-based AAL technologies from their non-visual counterparts (eg, pendant alarms), such as the ability to intervene in hazardous situations without manual intervention by the user, were imperative to the acceptance decisions of some older adults (74). Acceptance was also heightened when the technology was seen as being able to foster social relationships by enabling remote communication with others (13,28,51,53,65,79,81–83,89).

Acceptance was also subject to the technology’s configuration. For example, acceptance was enhanced if older adults were reassured that camera-acquired information would be transmitted only to authorized individuals such as trusted family members, caregivers, or healthcare professionals (24,53,59,63,65,72,75,88,90). The use of identity-redacting privacy filters also increased acceptance (19,63,71,77–79,88). In fact, this was a key precondition to acceptance for many: “I think it’s essential [that] it’s blurred, you can’t recognize the person” (77); “Now, if it showed who you are—that would be different” (88).

### Social/Professional Role and Identity

Acceptance was diminished insofar as older adults did not think that they needed the technology (13,21,27,53,62,65,72,78,88–90). Many professed to have “young” (89) and “healthy” (72) dispositions with “no aging-related difficulties” (72) that would warrant the use of camera-based AAL technologies.

Acceptance was also mitigated by the technology’s stigmatizing quality, which was often taken to reify undesirable notions of infirmity and dependence (13,19,20,56,63,64,77,88–90). Monitored spaces were frequently experienced as institutionalizing, with cameras seen as encroaching on older adults’ autonomy to do as they so pleased at home (13,21,56,60,63–65,69,72,73,83,88–90). Rejection or discontinuation of use was common among those who felt policed by the technology: “Now, if I go lie down and have my little granny nap, that thing up there is going to tell somebody on the other end I’m having a rest” (65). Others feared becoming too dependent on technology (13,21,69,83), with some alluding to images of a dystopian, technology-suffused world (23). Paradoxically, although camera-based AAL technologies were typically advocated as tools for enhancing independence, the opposite belief was common among older adults who worried that the technology would place them under undue control by their family members (56,60,63,65,73). For instance, 2 studies found that older adults were wary about decisional interference by family members who might, on the basis of information obtained through technology, decide on their early transition to long-term care (56,65).

Interestingly, some older adults who denounced their own need for camera-based AAL technologies were nevertheless enthused about the technology’s usefulness for *other* people—for example, individuals perceived as “worse off,” “more infirm,” or having “more difficulty” than themselves (76). This self-other distinction in the perceived need for the technology was a recurrent barrier to acceptance (13,27,65,72,76,88,90). Others drew on so-called “technophobic” (19,89) identities to reject the prospect of any technological interference in their lives: “No, I don’t like technology. I’m so old-fashioned. I don’t want anything to do with technology” (76).

Across the board, camera-based AAL technologies were better received by older adults who perceived themselves to be in need of care (13,20,21,23,24,27,28,41,51–53,56,62,64,65,67,69,72,74,75,78,79,87,88,90). Some were even reticent to exclude cameras from sensitive spaces such as the bathroom and bedroom due to elevated concerns about the heightened potential for falls to occur in these areas (41,67,76).

Interestingly, the presence of an objective need for the technology was not a stringent precondition to acceptance. Rather, a perceived need for the technology could be induced to benefit acceptance. For example, one study found that older adults were reluctant to use camera-based AAL technologies in their homes, opting instead for non-visual AAL technologies. However, when asked to imagine themselves in a dangerous situation (eg, experiencing a fall) at home, older adults exhibited a reversal of preference, preferring camera-based over non-visual AAL technologies (62).

### Environmental Context and Resources

Camera-based AAL technologies that were seen as unaffordable (13,20,28,51,52,57,58,63,65,66,69–73,76,82,83,85,87,89), obtrusive (19,20,52,53,62–64,67–69,74,77,82,84,87,89,90), or difficult to use (13,19,20,28,51–53,56,57,63,66,68,70,72,74,81,85,89) were less frequently accepted. Some older adults noted that their homes were structurally unsuited to accommodate complex camera infrastructure (13,27,58,66,67,85). Testifying the significance of material considerations, one study found that ownership of the technology was positively predicted by income level and home square footage (58). Additionally, acceptance was diminished where the technology was seen to integrate poorly into older adults’ daily lives, which often featured cherished routines that were neither expendable nor malleable (20,28,53,74). Additionally, older adults who had existing AAL technologies in their homes saw camera-based AAL technologies as redundant and thus less acceptable (27,72).

Conversely, acceptance was heightened when the technology was deemed unobtrusive and easy to use, and considerate of the functional limitations potentially faced by older users (19,20,51,53,57,62–64,66–72,74–77,79,81,82,87,89,90). Desires for cameras to be “as invisible as possible” (69) abounded in the data, with some advocating for cameras to be unobtrusively “hidden behind mirrors” rather than “hanging from the ceiling” (69), with the number of visible devices and cables kept to a minimum (74). Acceptance was also more readily extended when camera-based AAL technologies were designed in such ways as to be easily used by older adults with cognitive impairment or sensory deficits (74), with automated functions that circumvented the need for manual button presses (81).

### Barriers and Facilitators With Moderate Importance

Five TDF domains contained moderately important determinants of acceptance, being represented in over a third of identified codes. These were: *social influences* (8.86%), *reinforcement* (8.36%), *beliefs about capabilities* (5.34%), *memory, attention, and decision processes* (4.73%), and *emotion* (4.53%).

### Social Influences

To many, camera-based AAL technologies were neither a viable nor acceptable alternative to human caregiving (13,19,21,23,28,70–72,82,83,85,87,89). Many were concerned that the technology would create unnecessary burdens for their caregivers by triggering unwarranted alerts (28,56,63,71,72,89,90) or by inundating caregivers with superfluous details about their personal lives: “They’ve got their own problems and their families” (56). The presence of existing care support networks (eg, spousal support) also led to reduced acceptance (13,57,65,90).

Conversely, some older adults saw camera-based AAL technologies as harboring burden-relieving properties due to their ability to remotely reassure caregivers about their wellbeing (13,51,56,60,65,72,82,88,92). Profoundly, some camera-based AAL technologies were seen as social counterparts in and of themselves. For example, social robots were often conceived as engaging companions—that is, “a friend” (84,86)—with whom older adults could interact to alleviate loneliness. Also important was the presence of ongoing social or technical support to help older adults navigate unfamiliar technology (19,51–53,56,63,65,66,70,71,74,84,87). Social incentives were also influential facilitators, as many were more willing to use technology if advised to do so by trusted healthcare professionals or family members (27,63,70,89), or if the technology was widely adopted in the community (64,87,90).

### Reinforcement

A prominent pattern in the literature was that acceptance was contingent on past experience. For instance, acceptance was attenuated among those who did not (vs did) have prior experience with assistive technology or technology more generally (13,20,51,57,58,65,66,70,76,80,84,87,89). Some emphasized that they did not “grow up” with technology and thus lacked the necessary experience to use the technology competently (13,76). Relatedly, one study demonstrated that existing owners of smart home technology were significantly more likely to be brisk adopters of camera-based AAL technologies compared to non-owners (58). Acceptance was also tempered among those without a history of falls, disability, or cognitive impairment (54,58,59,67), as well as those who reported being more independent in their daily lives (57,58).

Conversely, acceptance was facilitated by prior technological experience and ownership (20,56–58,65,67,69,76,77,79,80,85,87,89). This is apparent in the quote: “Maybe because I’m used to my motion detector alarm system, I’m used to having a camera that monitors my movements” (77). Illuminating longitudinal findings demonstrated that acceptance increased over time as older adults familiarized themselves with the technology (77,79,89). With time, they “don’t even know that [the technology] is there […] it has become part of the scenery” (79).

Acceptance was also elevated among older adults who had (vs did not have) a history of health-related adversities such as prior fall incidents (56–58,74,88,90), heart attack (76), or stroke (69). This is evident in one statement: “If you had told me 2 months ago [about these technologies] I’d say who needs it, but after what I have been through, I see the benefits” (90). Acceptance was also heightened among those with (vs without) disability (59) or psychogeriatric conditions such as mild cognitive impairment (54) or dementia (68). Being presently in receipt of in-home care, such as nurse visits, also facilitated acceptance (41,69).

### Beliefs About Capabilities

Acceptance was attenuated among older adults who lacked the self-efficacy to use the technology (13,65,69,84). Some reported lacking the “digital confidence” (84) necessary to trek the learning curves associated with using the technology, whereas others felt that doing so was out of their comfort zones (65).

Quintessential to many acceptance decisions was the ability to maintain control over the technology’s operation, including where, when, how, and what kinds of recordings take place, as well as to whom data and under what conditions data are transmitted (13,24,28,56,62–66,69,71–73,75,77,78,83,88–90). Older adults reported feeling “less spied on” if the cameras were configured to only activate upon motion detection or only during specific, user-specified times in the day (77). Additionally, many expressed diminished privacy concerns if monitoring was conducted by family members or trusted professionals (24,59,88,90). Conversely, however, a prerequisite to acceptance for some older adults was the express ability to customize their data-sharing preferences and forbid access to family members, granting access to healthcare professionals instead (65,73,89).

### Memory, Attention, and Decision Processes

Dominant in the literature was a temporal specificity in older adults’ willingness to accept the technology. Many saw no present need for the technology and deferred this need into the future instead (13,27,28,61,63,65,72,78,88–90). In one study, although an overwhelming majority (95.5%) of older adults agreed that camera-based AAL technologies would be useful in later life, only a minority (25.3%) ceded to its present-day usefulness. Consequently, even fewer (15.5 %) were willing to use the technology at this point in their lives (63).

Cost-benefit trade-offs pervaded older adults’ cognitive assessments of the technology. Although many steadfastly upheld their need for privacy when considering the prospect of camera-mediated surveillance, many were willing to give up this privacy for the technology’s perceived merits (13,19,23,24,27,28,64,65,67,69–72,74,75,78,79,90). For instance, privacy concerns were frequently superseded by the need to be reassured about one’s health and safety: “If you are to the point where you need the help […] then your privacy becomes secondary [and] your privacy has to go out the window” (24). Others waived their right to privacy if this was seen as the sole recourse to avoiding institutional care (23,24,28,65,72). As in one evocative quote: “If you tell me that being monitored at home is a real alternative to a retirement home, I would accept it” (23).

### Emotions

That cameras created disconcerting feelings of “being watched” (23,77) or “spied on” (13,77) by unwanted entities frequently prompted rejection. Prevalent in older adults’ disavowals of technology were allusions to “Big Brother” and fearful, anxious, or generally negative attitudes toward technology (13,21,59,69,71,74,76,80,85). One longitudinal assessment found that discontinued use of the technology was solely attributable to feeling uneasy and panicked when subject to camera-based surveillance (74). Where cameras were visible to others, what typically followed was pre-emptive rejection to avoid potential embarrassment or shame: “I haven’t told anyone about my incontinence problem, so I remained a little embarrassed” (77).

Notably, acceptance was elevated among those who feared being without help during emergencies (67,69,79). As intimated by one older adult: “If I fell at night, someone would know. I really do feel safer because I fear falling” (79).

### Barriers and Facilitators With Marginal Importance

Four TDF domains were identified as having a marginal influence on acceptance, being represented across approximately 5% of codes. These were: *knowledge* (2.32%), *goals* (1.31%), *optimism*, (0.91%) and *skills* (0.70%).

### Knowledge

Acceptance was hindered by older adults’ lack of knowledge and/or understanding of camera-based AAL technologies, or of technology in general (13,19,51,52,56,63,65,66,68–70,72,74,85,87). For instance, acceptance was diminished among those with poor digital literacy (51,70), those who did not understand the technology’s purposes and functions (19,65,85), and those who were not aware of the technology’s market availability (52,69,85). Many professed that they would be more accepting of the technology if they simply knew more about it: “You need to perhaps have a session in places like [senior centers] and you say, well, these are the benefits” (65). For some, acceptance was contingent on having been sufficiently informed about the technology’s privacy policies and having the necessary knowledge on how to protect their personal data (56,72).

### Goals

The desire to age in place and avoid institutionalization also facilitated acceptance (24,28,65,71,72,74,76,87). Many older adults wished to remain in their own homes and ceded that using camera-based AAL technologies may be a primary recourse for reaching this goal. This is notable in the impassioned statement: “Nursing home? Don’t you ever tell me of that word, it’s like a dirty word to me” (71).

### Optimism

Acceptance was impeded by older adults’ tendencies to harbor seemingly unrealistic levels of optimism about their wellbeing, where many believed themselves to have better health than was objectively the case (13,27,65,72,89). One study found that older adults with pulmonary conditions, degenerative diseases, mobility impairments, or established fall histories nevertheless remained hesitant to use the technology: “I don’t have any balance anymore, I have a plate in this leg […] and it makes me wobbly sometimes [yet] I’d have to be very dependent on my cane [to use the technology]” (27). Another study found that even those with definitive health complications such as multimorbidity and prior stroke were inclined to wait until a later time—that is, when they “really needed” it—to use the technology (65).

### Skills

Older adults who lacked the skills needed to operate camera-based AAL technologies had less welcome dispositions toward the technology (13,19,52,66,70,72,76,84). Some expressed a desire for training and upskilling sessions, which were deemed important for those who lacked the technical repertoires to navigate the technology (19,52,66,70,84).

## Discussion

The results of this scoping review illuminate an array of barriers and facilitators to older adults’ acceptance of camera-based AAL technologies, which span 12 theoretically important domains of behavioral influence. The factors identified as having significant bearing on acceptance pertain not only to utilitarian issues concerning the technology’s usefulness and usability but also relate to psychological, social, and environmental facets.

### Barriers

Reasons for rejection of camera-based AAL technologies were predominated by concerns regarding the technology’s privacy implications. Across the board, being subject to camera-based surveillance was deemed untenable, as cameras often evoked feelings of fear, anxiety, or shame that prompted rejection or discontinuation. The ways in which cameras constrained older adults’ behaviors at home also compelled rejection. To many, having privacy was consonant with an upheld ability to do as they so pleased and to be excluded from the controlling gaze of cameras, professionals, and family members alike.

The retrieved data also confirmed the TAM’s empirical provenance, as camera-based AAL technologies were frequently rejected on account of being seen as difficult to use—this concern was typified in older adults who lacked prior technological experience or who lacked the self-efficacy to use the technology. These findings are consistent with established literature documenting strong positive linkages between experience with technology and technology self-efficacy among older adults, with both contributing to technology acceptance (93). Complex devices that were misaligned with older adults’ physical and cognitive faculties were typically unwelcome, as were bulky, unreliable assemblages that did not integrate into their lifestyles and routines.

Acceptance depended not only on the technology’s utilitarian value but also on how it fits within the occupied or desired identities of older adults. Although self-ascribed “technophobes” (19,89) reviled exposure to the alienating technology, others saw the technology as redundant on account of having existing social supports at home, or having purportedly “young” (89) and “healthy” (72) dispositions. Accordingly, many preferred to defer the use of the technology into the future when they “really needed it” (65), or onto other “more infirm” (76) individuals. Crucially, this lack of perceived need was on occasion misguided, as even older adults with categorically verifiable impairments or a history of adverse health did not feel that they needed the technology.

These findings cohere with established literature on the problematic self-presentational implications of assistive technology use (94,95). According to Jones (96), by reneging on the need for assistive technology, older adults effectively position themselves in a minority of older individuals with atypical vitality and health to reinforce desired “not-old” identities. The present analysis reveals that this unrealistic optimism serves a similar rhetorical benefit in the context of decisions surrounding camera-based AAL technologies. Specifically, by espousing counter-narratives about being “blessed with good health all [their] life” (27) despite having various aging-related health complications, older adults can delegitimize stereotyping narratives about “oldness” and construct more desirable identities for themselves instead.

Interpersonal factors also formed influential barriers to acceptance. Camera-based AAL technologies were frequently seen as unfit for subsuming necessarily human caregiver roles. Many were worried about the potential for system-generated alerts to inconvenience or overwhelm their family members and caregivers. Paradoxically, those who had existing social support saw the technology as superfluous and thus less acceptable.

### Facilitators

The accumulated findings point to several strategies to enhance older adults’ acceptance of camera-based AAL technologies. Centrally, the use of anonymizing privacy filters and ensuring secure processes in data acquisition, storage, and transmission promoted acceptance. Older adults were more accepting of the technology if they could designate access to camera-acquired information to particular individuals of their choosing: some preferred that their data be made available to family members, whereas others forbade the same access and preferred that their data were accessible only to healthcare professionals. Acceptance was also heightened when older adults were granted decisional control over the technology’s form and function, such as when older adults could decide where, when, how, and what kinds of data were captured by cameras. Counterintuitively, some saw the technology as augmenting rather than detracting from their privacy when considered against the alternative of institutionalization. Emphasizing the technology’s privacy-enhancing quality may thus represent a useful strategy to enhance acceptance.

Conditional reasoning abounded in older adults’ acceptance decisions, with many being willing to trade their privacy for perceived benefits in health, safety, and independence. Privacy concessions were deemed necessary insofar as older adults saw a genuine need for the technology. This was especially evident among those with a history of adverse health or those who feared being helpless during emergencies. Presumably, when juxtaposed against previous adversity, camera-based AAL technologies were conceived as valuable additions to older adults’ lives rather than as superfluous appendages: “Just think how important this would have been when that [fall] happened. It would have been better if we had this all the time” (88). Older adults were also typically willing to sacrifice their privacy if this assured delays to or avoidance of transitions to residential care.

Factors within older adults’ environmental and social contexts also emerged as important facilitators. The use of unobtrusive design, such as where cameras were “hidden behind mirrors” (69) or miniaturized (77), improved acceptance. These findings cohere with studies documenting linkages between the visibility of assistive technologies and their stigmatizing quality (97). It stands to reason, then, that cameras that were “out of sight [were also] out of mind” (76), allowing older adults to go about their daily lives without the self-consciousness that would typically entail in the use of AAL technologies. Additionally, subsidized, low-complexity, and easy-to-use systems were better received than their more expensive or cumbersome counterparts (70,71). The provision of training and upskilling sessions to enhance older adults’ digital self-efficacy may therefore benefit acceptance.

Exploiting social levers may also represent an influential acceptance-promoting strategy. Camera-based AAL technologies were better received if they were seen to relieve caregiving burdens rather than as extraneous stressors. Older adults were also more receptive to the technology if they saw it as an aid to fostering social relationships or if they deemed the technology as a social counterpart in and of itself, as was frequently the case with social robots. Acceptance was also sensitive to prevailing social norms: the technology was better accepted if it was believed to be widely disseminated (ie, a *descriptive norm* of acceptance existed (98)) or if adoption was actively encouraged by important others (ie, an *injunctive norm* of acceptance existed (98)). These findings accord with the postulates of the social norms approach to behavior, which suggests that people often behave as others do and in line with what is seen as socially appropriate due to an ingrained preference for conformity and social approval (99). Relatedly, because behavior that is widespread is also seen as “normal” (99), social norms may have powerful de-stigmatizing effects that may benefit acceptance. For example, if self-image can be preserved by bringing to the fore the supposed discrepancies between who the technology is meant for—that is, the “older [and] more infirm” (76)—and who older adults believe themselves to be—that is, “still young [and] capable” (89), messages that convey the popularity and prevalence of camera-based AAL technologies may attenuate the technology’s stigmatizing quality and thus increase its acceptability to older adults.

### Practical Implications

The results of this review have important implications for the design, development, and dissemination of camera-based AAL technologies. Acceptance-facilitating strategies include the use of anonymizing privacy filters and granting decisional control to older adults over facets relating to the technology’s form and function. This granting of control may assuage older adults’ concerns about overreliance on technology—for instance, the ability to turn cameras on or off as they so wish should allow older adults to retain a semblance of control over aspects of their lives that they prefer to be left untouched by technology. Where possible, cameras should be concealed or miniaturized to attenuate their stigmatizing quality, with additional benefits realized in terms of potentially less costly and resource-demanding assemblages. The socially contingent nature of older adults’ acceptance decisions also suggests promising directions for optimizing the technology’s dissemination. Rather than marketing the technology as prosthetic compensation for aging-related “deficits,” practitioners might better promulgate (where verifiable) the technology’s prevalence and popularity, which should mitigate the stereotyping lens normally placed on users of assistive technology. Dissemination strategies might also emphasize the burden-relieving and relationship-building properties of the technology for older adults and their care networks. Efforts should also focus on better aligning older adults’ technological repertoires with the technology’s pragmatic requirements. Besides developing more simplistic and learnable systems, developers could capitalize on the remunerative potential of education and upskilling to augment older adults’ digital repositories and help overcome residual fears or anxieties surrounding the technology.

### Strengths and Limitations

This review contributes to the growing body of literature on the factors influencing older adults’ acceptance of assistive technology. To the best of our knowledge, it is the first to focus on camera-based modalities of AAL and is eminent in its use of psychological theory to synthesize the barriers and facilitators to acceptance.

Nonetheless, several limitations warrant consideration. Studies were not assessed for quality as quality appraisals are beyond the scope of scoping reviews (36); this precludes an unbiased interpretation of the results. Furthermore, the importance of barriers and facilitators cannot be confidently verified on the basis of our content analysis, which used coding frequency as a proxy for importance. This is because our review considered all study designs, and barriers and facilitators were extracted irrespective of the strength or precision of relationships. As such, TDF domains that were sparsely populated may nevertheless have a potent influence on acceptance. Researchers should therefore exercise prudence when drawing conclusions about which barriers or facilitators to target based on their ranked importance presented in this review.

Of important note here is that the absence of barriers and facilitators mapped to the *intentions* and *behavioral regulation* domains does not necessarily preclude their influence on acceptance. According to the developers of the TDF, *goals* refer to mental representations of outcomes or end-states that individuals strive toward, whereas *intentions* concern a conscious decision to behave or act in a certain way (32). However, researchers assessing the TDF’s discriminant content validity have highlighted that goal-related constructs can be classified into both domains, making it especially difficult to code discriminately between the 2 (100). Indeed, previous TDF-based syntheses of behavioral barriers and facilitators have faced difficulties in achieving inter-coder consensus when coding to the *intentions* and *goals* domains (48). The decision rules applied during the coding phase in this scoping review were such that older adults’ professed desires to avoid the transition to long-term care were most suitably mapped to the *goals* rather than the *intentions* domain, as these desires were deemed as relating more to an explicit, pinpointable end-state of aging in place. Nonetheless, it is entirely likely that mental representations of an aging in place goal manifest simultaneously with the intention to behave in ways that would prevent institutionalization. The dearth of coding to the *intentions* domain may therefore be an artifact of the conceptual parsimony sought in the review process rather than reflect any genuine theoretical irrelevance of the construct.

Tangentially, the absence of barriers and facilitators coded to the *behavioral regulation* domain is likely attributable to this review’s focus on the precursors to acceptance rather than the factors involved in maintaining the already established usage of the technology. This is because, whereas the current study is focused on the antecedents to acceptance, factors that are aptly mapped to the *behavioral regulation* domain better relate to the self-regulatory means with which individuals attempt to manage ongoing behavior or behavior change (32). Therefore, although this review has uncovered factors pertinent to the pre-implementation stage of using camera-based AAL technologies, factors mappable to the *behavioral regulation* domain may very well be significant in postimplementation stages where older adults might attempt to maintain long-term use of the technology.

### Future Directions

The results suggest several promising avenues for future research. As evidenced by the scarcity of included studies, research on older adults’ acceptance of camera-based AAL technologies is limited. Indeed, of the total number of potentially eligible articles retrieved from the database searches, only a minority (1.27%) were interested in the influences on older adults’ acceptance of AAL technologies. A considerable number of studies were excluded from the review due to an overtly utilitarian focus on more technical aspects such as the technology’s fall detection accuracy. Given that successful assistive technology deployments are contingent on users’ acceptance of the technology (11), future enquiries into the determinants of acceptance are warranted.

Where studies evaluated factors relevant to the acceptance of camera-based AAL technologies, none attempted to ground the identified factors in relevant theory. Given the centrality of behavior and behavior change in technology acceptance (11), this lack of theoretical posturing may account for the presently sluggish dissemination of AAL technologies. This is because, in the absence of theory, the design and dissemination of such technology may be mismatched to barriers and fail to leverage facilitators relevant to acceptance. Future studies would therefore do well to emulate this theory-driven approach to identify the determinants of AAL technology acceptance, with concurrent elaboration on appropriate acceptance-facilitating behavior change techniques.

A point worthy of critical reflection here is that some older adults vehemently rejected the use of camera-based AAL technologies, typically on account of their intrusive quality, their stances unswayed by elaboration on the technology’s purported benefits. Although this state of affairs brings into question the legitimacy of advancing research on camera-based AAL technologies, evidence on the myriad ways in which other older adults concede to the use of the technology—for instance, where such use is seen as preventing an otherwise secured transition to residential care—suggests that there is empirical and practical value in understanding the various levers to change acceptance, at least in persuadable cohorts of older adults. Such research is important to ensure that the healthspan and lifespan-promoting value of camera-based AAL technologies are translated from rhetoric to reality.

## Conclusion

This review presents a synthesis of the barriers and facilitators to older adults’ acceptance of camera-based AAL technologies. Results revealed a range of physical, psychological, social, and environmental antecedents to acceptance that span a theoretical remit far wider than that which has been portrayed in traditional technology acceptance models. Technology developers might utilize these findings to inform the development and dissemination of more acceptable—and thus more widely used—camera-based AAL technologies. Future studies might leverage the present findings to delineate appropriate behavior change techniques, targeted at theoretically robust barriers and facilitators, that can be used in acceptance-facilitating interventions. Such double-pronged strategies may best ensure the widespread diffusion of, and older adults’ sustained engagement with, camera-based AAL technologies.

## Supplementary Material

igae100_suppl_Supplementary_Tables

## Data Availability

This study was not preregistered. The “data” are a scoping review that has all the “data” in tables for articles included in the review. The search strategy used in the review is available in the Supplementary Material.
