# Supplementary material for: Barriers and Facilitators to Older Adults’ Acceptance of Camera-Based Active and Assisted Living Technologies: A Scoping Review
Source: Innov Aging. 2024 Nov 29;9(2):igae100. doi: 10.1093/geroni/igae100 (PMC11833315; doi:10.1093/geroni/igae100)
Supplement: igae100_suppl_Supplementary_Tables [file igae100_suppl_supplementary_tables.docx]

***Innovation in Aging* Supplementary Material: Tham et al. Barriers and facilitators to older adults’ acceptance of camera-based active and assisted living technologies: A scoping review.**

Supplementary Table 1: MEDLINE search strategy.

| S1 | AB (aged OR ageing OR aging OR elder* OR frail* OR geriatr* OR gerontol* OR “later in life*” OR “later life*” OR “old age” OR “old* adult*” OR “old* individual*” OR “old* people*” OR “old* person*” OR “old* vulnerable” OR retire* OR senescent OR senile OR senior* ) OR TI (aged OR ageing OR aging OR elder* OR frail* OR geriatr* OR gerontol* OR “later in life*” OR “later life*” OR “old age” OR “old* adult*” OR “old* individual*” OR “old* people*” OR “old* person*” OR “old* vulnerable” OR retire* OR senescent OR senile OR senior* ) |
| --- | --- |
| S2 | AB (abandon* OR accept* OR adhere* OR adopt* OR assimilat* OR attitud* OR belief* OR compliance OR comply OR consideration* OR expect* OR experience* OR fear* OR feel* OR integrat* OR intent* OR non-use OR nonuse OR opinion* OR perceive* OR perception* OR perspective* OR preference* OR reason* OR reject* OR satisf* OR view*) OR TI (abandon* OR accept* OR adhere* OR adopt* OR assimilat* OR attitud* OR belief* OR compliance OR comply OR consideration* OR expect* OR experience* OR fear* OR feel* OR integrat* OR intent* OR non-use OR nonuse OR opinion* OR perceive* OR perception* OR perspective* OR preference* OR reason* OR reject* OR satisf* OR view*) |
| S3 | AB (“age in place” OR “age-in-place” OR “ageing at home” OR “ageing from home” OR “ageing in place” OR “ageing-in-place” OR “aging at home” OR “aging from home” OR “aging in place” OR “aging-in-place” OR “at home” OR at-home OR “community dwelling” OR “community-dwelling*” OR domicile OR dwelling* OR house* OR home* OR home-based OR home-environment* OR “home environment*” OR “in home” OR in-home OR “private home*” OR residenc* OR residential OR “retirement communit*” OR “retirement home*” OR “retirement village*”) OR TI (“age in place” OR “age-in-place” OR “ageing at home” OR “ageing from home” OR “ageing in place” OR “ageing-in-place” OR “aging at home” OR “aging from home” OR “aging in place” OR “aging-in-place” OR “at home” OR at-home OR “community dwelling” OR “community-dwelling*” OR domicile OR dwelling* OR house* OR home* OR home-based OR home-environment* OR “home environment*” OR “in home” OR in-home OR “private home*” OR residenc* OR residential OR “retirement communit*” OR “retirement home*” OR “retirement village*”) |
| S4 | S1 AND S2 AND S3 |
| S5 | ((MH “Ambient Intelligence” OR AAL OR “activity recognition” OR “activity detection” OR “ambient assist*” OR ambient-assist* OR “ambient intelligence” OR “ambient intelligent” OR AmI OR “assist* living” OR eldercare OR “independent living” OR monitor* OR “motion detect*” OR “motion recognit*” OR “smart building*” OR “smart home*” OR “smart house*” OR “smart residence*” OR surveil* OR “ubiquitous comput*” OR “ubiquitous monitor*”) N2 (technolog* OR system* OR device* OR application*) |
| S6 | S4 AND S5 |

Supplementary Table 2: Data coding manual.

| **TDF domain and content** | **Constructs** | **Decision Rules** |
| --- | --- | --- |
| **Knowledge:** An awareness of the existence of something | Knowledge (including knowledge of condition): An awareness of the existence of something | Consider coding to this domain:   - Discussion relating to older adults’ knowledge and understanding (or lack thereof) of how to use camera-based AAL technologies. - Discussion relating to older adults’ awareness of the existence and/or market availability of camera-based AAL technologies.   Inappropriate coding to this domain:   - Discussion of personalised accounts of hypothetical behaviour - e.g., statements such as “I would educate myself on how to use the technology” should be coded at “Behavioural Regulation” instead. |
|  | Procedural knowledge: Knowing how to do something |  |
|  | Knowledge of task environment: Knowledge of the social and material context in which a task is undertaken |  |
| **Skills:** an ability or proficiency acquired through practice | Skills: An ability or proficiency acquired through training and/or practice | Consider coding to this domain:   - Discussion about the skills (or lack thereof) employed by older adults in order to use camera-based AAL technologies. - Discussion about training (to be) undertaken by older adults in order to use camera-based AAL technologies.   Inappropriate coding to this domain:   - Discussion relating to older adults’ confidence in their ability to use camera-based AAL technologies should be coded at “Beliefs about capabilities” instead. |
|  | Skills development: The gradual acquisition or advancement through progressive stages of an ability or proficiency acquired through training and practice |  |
|  | Competence: One’s repertoire of skills, and ability especially as it is applied to a task or set of tasks |  |
|  | Ability: Competence or capacity to perform a physical or mental act. Ability may be either unlearned or acquired by education and practice |  |
|  | Interpersonal skills: An aptitude enabling a person to carry on effective relationships with others, such as an ability to cooperate, to assume appropriate social responsibilities or to exhibit adequate flexibility |  |
|  | Practice: Repetition of an act, behaviour, or series of activities, often to improve performance or acquire a skill |  |
|  | Skills assessment: A judgement of the quality, worth, importance. Level or value of an ability or proficiency acquired through training and practice |  |
| **Social/Professional role and identity:** A coherent set of behaviours and displayed personal qualities of an individual in a social or work setting | Professional identity: The characteristics by which an individual is recognised relating to, connected with, or befitting a particular profession | Consider coding to this domain:   - Discussion relating to older adults’ perceived need (or lack thereof) for camera-based AAL technologies. - Discussion relating to older adults’ belief about the stigmatising qualities of camera-based AAL technologies. - Discussion relating to how older adults’ self-identity - e.g., as “old-fashioned” individuals - impacts their acceptance of camera-based AAL technologies. - Discussion of older adults’ belief that using camera-based AAL technologies threatens their autonomy or dignity.   Inappropriate coding to this domain:   - Statements relating to social relationships that influence older adults’ acceptance of camera-based AAL technologies – e.g., descriptions of older adults’ need for social approval from peers or family members and how this impacts their acceptance decisions should be coded at “Social Influences” instead. |
|  | Professional role: The behaviour considered appropriate for a particular kind of work or social position |  |
|  | Social identity: The set of behavioural or personal characteristics by which an individual is recognisable [and portrays] as a member of a social group |  |
|  | Identity: An individual’s sense of self defined by a) a set of physical and psychological characteristics that is not wholly shared with any other person and b) a range of social and interpersonal affiliations (e.g., ethnicity) and social roles. |  |
|  | Professional boundaries: The bounds or limits relating to, or connected with a particular profession or calling |  |
|  | Professional confidence: an individual’s belief in his or her repertoire of skills and ability especially as it is applied to a task or set of tasks. |  |
|  | Group identity: the set of behavioural or personal characteristics by which an individual is recognisable [and portrays] as a member of a group |  |
|  | Leadership: The processes involved in leading others, including organising, directing, coordinating, and motivating their efforts toward achievement of certain group or organisation goals |  |
|  | Organisational commitment: An employee’s dedication to an organisation and wish to remain part of it. Organisational commitment is often described as having both an emotional or moral element and a more prudent element |  |
| **Beliefs about capabilities:** Acceptance of the truth, reality, or validity about an ability, talent, or facility that a person can put to constructive use | Self-confidence: Self-assurance or trust in one’s own abilities, capabilities, and judgement | Consider coding to this domain:   - Descriptions of older adults’ sense of aptitude for, or confidence in, using camera-based AAL technologies (i.e., perceived self-efficacy). - Descriptions of older adults’ perceived ability to have control over the functioning and/or operations of camera-based AAL technologies (e.g., turning cameras on/off, deciding where, when, and how recording takes place, and to whom information should be transmitted). - Descriptions of older adults’ belief that they have control over the decision of whether to have camera-based AAL technologies installed in their home.   Inappropriate coding to this domain   - Descriptions of older adults’ beliefs about the capabilities of other individuals (e.g., family members, informal/formal caregivers) to use camera-based AAL technologies. |
|  | Perceived competence: An individual’s belief in her or her ability to learn and execute skills |  |
|  | Self-efficacy: An individual’s capacity to act effectively to bring about desired results, as perceived by the individual |  |
|  | Perceived behavioural control: an individual’s perception of the ease or difficulty of performing the behaviour of interest |  |
|  | Beliefs: The thing believed; the proposition or set of propositions held true |  |
|  | Self-esteem: The degree to which the qualities and characteristics contained in one’s self-concept are perceived to be positive |  |
|  | Empowerment: The promotion of the skills, knowledge, and confidence necessary to take great control of one’s life as in certain educational or social schemes; the delegation of increase decision-making powers to individuals or groups in a society or organisation. |  |
|  | Professional confidence: An individual’s beliefs in his or her repertoire of skills, and ability, especially as it is applied to a task or set of tasks. |  |
| **Optimism:** The confidence that things will happen for the best or that desired goals will be attained | Optimism: The attitude that outcomes will be positive and that people’s wishes or aims will be ultimately fulfilled | Consider coding to this domain:   - Descriptions of older adults’ unrealistic levels of optimism regarding their current health status and/or the reality of their ageing – e.g., descriptions of older adults’ belief that they are “younger” or “healthier” than they really are – and how this impacts their acceptance of camera-based AAL technologies.   Inappropriate coding to this domain:   - Description of other people’s (e.g., family members, healthcare professionals) levels of optimism about the effectiveness and/or usefulness of camera-based AAL technologies for older adults. |
|  | Pessimism: The attitude that things will go wrong and that people’s wishes or aims are unlikely to be fulfilled |  |
|  | Unrealistic optimism: the inert tendency for humans to over-rate their own abilities and chances of positive outcomes compared to those of other people |  |
| **Beliefs about Consequences:** Acceptance of the truth, reality, or validity about outcomes of a behaviour in a given situation | Beliefs: The thing believed; the proposition or set of propositions held true | Consider coding to this domain:   - Descriptions of older adults’ beliefs about the potential negative outcomes that may result from their use of camera-based AAL technologies - privacy infringements, technical issues (e.g., false alarms). - Descriptions of older adults’ beliefs about the positive outcomes that may result from their use of camera-based AAL technologies – increased health, wellbeing, safety, independence, longevity, etc. - Descriptions of older adults’ belief that camera-based AAL technologies confer little utility. - Descriptions of older adults’ belief about outcomes relating to the data that will be collected, processed, and transmitted by camera-based AAL technologies – e.g., whether images and/or recordings are unfiltered or undergo processing for enhanced privacy and confidentiality. - Note: outcomes can be theoretical or as a result of actual experience.   Inappropriate coding to this domain:   - Descriptions relating to an anticipated outcome of using camera-based AAL technologies that is based on the beliefs that older adults have about themselves – e.g., descriptions of older adults’ belief that camera-based AAL technologies will be of little to no utility to them because they see themselves as “young and healthy” should be coded to “Social/Professional Role and Identity” instead. |
|  | Outcome expectancies: Cognitive, emotional, behavioural, and affective outcomes that are assumed to be associated with future or intended behaviour. These assumed outcomes can either promote or inhibit future behaviours. |  |
|  | Characteristics of outcome expectancies: Characteristics of the cognitive, emotional, and behavioural outcomes that individuals believe are associated with future or intended behaviours and that are believed to either promote or inhibit these behaviours. These include whether they are sanctions/rewards, proximal/distal, valued/not valued, probable/improbable. Salient/not salient, perceived risks or threats. |  |
|  | Anticipated regret: A sense of the potential negative consequences of a decision that influences the choice made: for example, an individual may decide not to make an investment because of the feelings associated with an imagined loss |  |
|  | Consequents: An outcome behaviour in a given situation |  |
| **Reinforcement:** Increasing the probability of a response by arranging a dependent relationship, or contingency, between the response and a given stimulus | Rewards (proximal/distal, valued/ not valued, probable/improbable): Return or recompense made to, or received by a person contingent on some performance. | Consider coding to this domain:   - Descriptions of how older adults’ previous usage of (assistive) technology impacts their current acceptance of camera-based AAL technologies. - Descriptions of how older adults’ previous experiences (e.g., prior adverse health events) impact their current acceptance of camera-based AAL technologies   Inappropriate coding to this domain:   - Descriptions of outcomes that older adults expect to result from using camera-based AAL technologies that are not contingent rewards – e.g., descriptions of older adults’ belief that using camera-based AAL technologies will relieve their family members’ caregiving burdens should be coded to “Social Influences” instead. |
|  | Incentives: An external stimulus, such as condition or object, that enhances or serves as a motive for behaviour |  |
|  | Punishment: The process in which the relationship between the response and stimulus or circumstance results in the response becoming less probable; a painful, unwanted, or undesired event or circumstance imposed as a penalty on a wrongdoer |  |
|  | Consequents: An outcome of behaviour in a given situation |  |
|  | Reinforcement: A process in which the frequency of a response is increased by a dependent relationship or contingency with a stimulus |  |
|  | Contingencies: A conditional probabilistic relation between two events. Contingencies may be arranged via dependencies or they may emerge by accident |  |
|  | Sanctions: A punishment or other coercive measure, usually administered by a recognised authority, that is used to penalise and deter inappropriate or unauthorised actions. |  |
| **Intentions:** A conscious decision to perform a behaviour or a resolve to act in a certain way | Stability of intentions: ability of one’s resolve to remain in spite of disturbing influences | Consider coding to this domain:   - Descriptions of older adults’ personal intent, motivation, or inclination to use camera-based AAL technologies. - Note: Use of the 1^st^ person “I will”, “I would” are strong indications to consider coding at this domain. - Note: Indicators of intention should be explicit and not inferred. Statements should therefore directly reflect older adults’ intention and/or motivation, rather than the reasons underpinning this intention.   Inappropriate coding to this domain:   - Descriptions of how older adults choose between two or more alternatives in order to reach an intended outcome – e.g., descriptions of how older adults prioritise their ability to remain at home over and above the preservation of their privacy should be coded at “Memory, Attention, and Decision Processes” instead. |
|  | Stages of Change model: A model that proposes that behaviour change is accomplished through five specific stages |  |
|  | Transtheoretical model and stages of change: a five-stage theory to explain changes in people’s health behaviour. It suggests that change takes time, that different interventions are effective at different stages, and that there are multiple outcomes occurring across the stages |  |
| **Goals:** Mental representations of outcomes or end states that an individual wants to achieve | Goals (distal/proximal): Desired state of affairs of a person or system, these may be closer (proximal) or further away (distal) | Consider coding to this domain:   - Descriptions of older adults’ acceptance of camera-based AAL technologies in relation to a distinct and identifiable endpoint - e.g., descriptions of older adults’ decision to use camera-based AAL technologies in order to avoid institutionalisation should be coded here. This differs from “Intentions” where older adults may describe their resolve to use the technology without any specific reference to an endpoint.   Inappropriate coding to this domain:   - Descriptions of how older adults prioritise one anticipated outcome of using the technology over another may be more appropriately coded elsewhere, especially if no specific reference is made to target endpoints – e.g., statements relating to trade-offs made by older adults between the preservation of personal privacy and increased health and independence should be coded at “Memory, Attention, and Decision Processes” instead. |
|  | Goal priority: Order of importance or urgency of end state toward which one is striving |  |
|  | Goal/target setting: A process that establishes specific time-based behavioural targets that are measurable, achievable, and realistic |  |
|  | Goals (autonomous/controlled): The end state toward which one is striving: the purpose of an activity or endeavour. It can be identified by observing that a person ceases or changes their behaviour upon attaining this state; proficiency in a task to be achieved within a set period of time |  |
|  | Action planning: The action or process of forming a plan regarding a thing to be done or a deed |  |
|  | Implementation intention: The plan that one creates in advance of when, where, and how one will enact a behaviour |  |
| **Memory, Attention and Decision Processes:** The ability to retain information, focus selectively on aspects of the environment and choose between two or more alternatives | Memory: The ability to retain information or a representation of a past experience, based on the mental processes of learning or encoding retention across some interval of time, and retrieval or reactivation of the memory; specific information of a specific task | Consider coding to this domain:   - Descriptions of the cognitive cost-benefit analyses that older adults engage in when contemplating usage of camera-based AAL technologies. - Descriptions of cognitive processes involved when older adults choose between two or more alternative outcomes in relation to using camera-based AAL technologies – e.g., trade-offs between preserving privacy and ageing-in-place. - Descriptions of older adults’ decisions regarding the timing of camera-based AALT usage – e.g., descriptions of older adults’ willingness to use camera-based AAL technologies in the future but not now.   Inappropriate coding to this domain   - Descriptions of older adults’ beliefs about their own decisional control over whether to use camera-based AAL technologies should be coded at “Beliefs about Capabilities” instead. |
|  | Attention: A state of awareness in which the senses are focused selectively on aspects of the environment and the central nervous system is in a state of readiness to respond to stimuli |  |
|  | Attention control: The extent to which a person can concentrate on relevant cues and ignore all irrelevant cues in a given situation |  |
|  | Decision making: The cognitive process of choosing between two or more alternatives, ranging from the relatively clear-cut to the complex |  |
|  | Cognitive overload/tiredness: The situation in which the demands placed on a person by mental work are greater than a person’s mental abilities |  |
| **Environmental Context and Resources:** Any circumstance of a person's situation or environment that discourages or encourages the development of skills and abilities, independence, social competence, and adaptive behaviour | Environmental stressors: External factors in the environment that cause stress | Consider coding to this domain:   - Descriptions of the obtrusiveness (or lack thereof) of camera-based AAL technologies, as perceived by older adults. - Descriptions of the affordability (or lack thereof) of camera-based AAL technologies, as perceived by older adults. - Descriptions of the ease-of-use (or lack thereof) of camera-based AAL technologies, as perceived by older adults - Descriptions of the availability (or lack thereof) of resources (e.g., Internet) to facilitate usage of camera-based AAL technologies. - Descriptions of the affordability of camera-based AAL technologies. - Descriptions of how camera-based AAL technologies are seen as being able or unable to integrate with older adults’ existing lifestyles.   Inappropriate coding to this domain:   - Descriptions of the actions taken by older adults in order to secure the necessary resources required to use camera-based AAL technologies should be coded at “Behavioural Regulation” instead. |
|  | Resources/material resources: Commodities and human resources used in enacting a behaviour |  |
|  | Organisational culture/climate: A distinctive pattern of thought and behaviour shared by members of the same organisation and reflected in their language, values, attitudes, beliefs and customs |  |
|  | Salient events/critical incidents: Occurrences that one judges to be distinctive, prominent or otherwise significant |  |
|  | Person-environment interaction: Interplay between the individual and their surroundings |  |
|  | Barriers and facilitators: In psychological contexts, barriers/facilitators are mental, emotional or behavioural limitations/strengths in individuals or groups |  |
| **Social influences:** Those interpersonal processes that can cause individuals to change their thoughts, feelings, or behaviours | Social pressure: the exertion of influence on a person or group by another person or group | Consider coding to this domain:   - Descriptions of older adults’ preference for human-provided care and interaction compared to technologically mediated care and interaction. - Descriptions of older adults’ concern about how camera-based AAL technologies may create unnecessary burdens for their caregivers. - Descriptions of older adults’ belief that using camera-based AAL technologies will relieve the burdens faced by their caregivers and/or healthcare professionals. - Descriptions of older adults’ belief that using camera-based AAL technologies will allow them to build stronger social relationships or expand their social networks. - Descriptions of how older adults’ acceptance of camera-based AAL technologies is influenced by the opinions or behaviours of others - e.g., family members, community peers, healthcare professionals. - Discussion of how instructions and/or guidance from others impact older adults’ acceptance of camera-based AAL technologies – e.g., descriptions of how older adults accept the technology due to medical directives from their physician. - Descriptions of how camera-based AAL technologies (e.g., social robots) are seen as social companions.   Inappropriate coding to this domain:   - Descriptions of older adults’ belief that camera-acquired data will be transmitted only to certain authorised individuals within their social network (e.g., family members) should be coded at “Beliefs about Consequences” instead, as this relates more to the perceived consequences of using the technology. |
|  | Social norms: Socially determined consensual standards that indicate a) what behaviours are considered typical in a given context and b) what behaviours are considered proper in the context |  |
|  | Group conformity: The act of consciously maintaining a certain degree of similarity to those in your general social circles |  |
|  | Social comparisons: The process by which people evaluate their attitudes, abilities, or performance relative to others |  |
|  | Group norms: Any behaviour, belief, attitude, or emotional reaction held to be correct or acceptable by a given group in society |  |
|  | Social support: The apperception or provision of assistance or comfort to others, typically in order to help them cope with a variety of biological, psychological, and social stressors. Support may arise from any interpersonal relationship in an individual’s social network, involving friends, neighbours, religious institutions, colleagues, caregivers of support groups |  |
|  | Power: The capacity to influence others, even when they try to resist this influence |  |
|  | Intergroup conflict: Disagreement or confrontation between two or more groups and their members. This may involve physical violence, interpersonal discord, or psychological tension |  |
|  | Alienation: Estrangement from one's social group; a deep-seated sense of dissatisfaction with one's personal experiences that can be a source of lack of trust in one's social or physical environment or in oneself; the experience of separation between thoughts and feelings |  |
|  | Group identity: The set of behavioural or personal characteristics by which an individual is recognisable [and portrays] as a member of a group |  |
|  | Modelling: In developmental psychology the process in which one or more individuals or other entities serve as examples (models) that a child will copy |  |
| **Emotion:** A complex reaction pattern, involving experiential, behavioural, and physiological elements, by which the individual attempts to deal with a personally significant matter or event | Fear: An intense emotion aroused by the detection of imminent threat, involving an immediate alarm reaction that mobilises the organism by triggering a set of physiological changes | Consider coding to this domain:   - Descriptions of the emotions experienced or anticipated by older adults in relation to being monitored by cameras – e.g., anxiety, fear, anger, etc. - Descriptions of the emotions experienced or anticipated by older adults in relation to being seen by others as users of camera-based AAL technologies – e.g., embarrassment, shame, humiliation, etc. - Descriptions of how fearful, anxious, or negative attitudes towards technology diminishes older adults’ acceptance of camera-based AAL technologies. - Descriptions of a fear of falling that compels older adults’ acceptance of camera-based AAL technologies.   Inappropriate coding to this domain:   - Descriptions of how other people’s emotions influence older adults’ acceptance of camera-based AAL technologies – e.g., descriptions of how older adults use camera-based AAL technologies because they do not want their children to feel worried should be coded at “Social Influences” instead. |
|  | Anxiety: A mood state characterised by apprehension and somatic symptoms of tension in which an individual anticipates impending danger, catastrophe, or misfortune. |  |
|  | Affect: An experience or feeling of emotion, ranging from suffering to elation, from the simplest to the most complex sensations of feelings, and from the most normal to the most pathological emotional reactions. |  |
|  | Stress: A state of physiological or psychological response to internal or external stressors |  |
|  | Depression: A mental state that presents with depressed mood, loss of interest or pleasure, feelings of guilt or low self-worth, disturbed sleep or appetite, low energy, and poor concentration |  |
|  | Positive/negative affect: The internal feeling/state that occurs when a goal has/has not been attained. A source of threat has/has not been avoided, or the individual is/is not satisfied with the present state of affairs |  |
|  | Burn-out: Physical, emotional, or mental exhaustion, especially in one’s job or career, accompanied by decreased motivation, lowered performance and negative attitudes towards oneself and others |  |
| **Behavioural Regulation:** Anything aimed at managing or changing objectively observed or measured actions | Self-monitoring: A method used in behavioural management in which individuals keep a record of their behaviour, especially in connection with efforts to changes or regulate the self; a personality trait reflecting an ability to modify one’s behaviour in response to a situation | Consider coding to this domain:   - Descriptions of the self-regulatory strategies employed by older adults that are aimed at facilitating their usage of or sustained engagement with camera-based AAL technologies. - Descriptions of the actions taken by older adults in order to secure the necessary resources to facilitate their own usage of or sustained engagement with camera-based AAL technologies.   Inappropriate coding to this domain:   - Descriptions of older adults’ belief that camera-based AAL technologies can help them to regulate their own behaviour – e.g., descriptions of older adults’ belief that using camera-based AAL technologies can facilitate their self-management of chronic disease should be coded at “Belief about Consequences” instead, as this relates more to the perceived consequences of using the technology. |

Note. AAL = active and assisted living; TDF = Theoretical Domains Framework.

Supplementary Table 3: Characteristics of included studies.

| **Authors** | **Country** | **Study objective** | **Study design** | **Method** | **Study participants** | **n of older adult sample** | **n Female of older adult sample (%)** | **Mean age of older adult sample** | **Type of camera-based AAL technology under evaluation** | **Context of evaluation of camera-based AAL technology** |
| --- | --- | --- | --- | --- | --- | --- | --- | --- | --- | --- |
| Albina & Hernandez (2018) | Philippines | To provide an initial understanding of the perceived needs, benefits and barriers to assistive technologies | Quantitative | Questionnaires | Older adults aged 60 and above | 118 | 37 (39.8%) | n/a | Standard camera | Private home |
| Alkhatib et al. (2021) | Australia | To gain a better understanding of what older adults who are ageing in place perceive as privacy problems in the use of aged care monitoring devices | Qualitative | Interviews and focus groups | Older adults aged 65 and above | 15 | 10 (66.7%) | 77.5 | Not described – participants were free to conjure images of “aged care monitoring devices”, including (but not limited to) camera-based AAL technologies | Private home |
| Alsulami et al. (2016) | United Kingdom | To explore the barriers and challenges of AAL technology adoption in Saudi Arabia and to provide a quantitative analysis based on the results of a survey | Quantitative | Questionnaires | Older adults aged 60 and above | 194 | 70 (36.1%) | n/a | Standard camera | Private home |
| Arthanat et al. (2019) | United States | To examine ownership of smart home technology by older adults, their readiness to adopt smart home technology, and identify the client factors relating to adoption | Quantitative | Questionnaires | Older adults aged 65-95 | 444 | 303 (68.1%) | 70.7 | Standard camera | Private home |
| Arthanat et al. (2020) | United States | To examine smart home technology ownership of older adults and its causal pathways with demographics, health and functioning, home safety and information communication technology use | Quantitative | Questionnaires | Older adults aged 65-95 | 447 | 306 (68.5%) | 70.9 | Standard camera | Private home |
| Beach et al. (2009) | United States | To examine the acceptability of sharing information through use of camera-based AAL technologies; To examine the acceptability of video-based recording of behavior; To examine whether age, disability, and their interaction are related to attitudes about recording and sharing health-related information in the context of quality-of-life technology; To examine whether actual use of assistive devices is related to privacy attitudes. | Quantitative | Questionnaires | Older adults aged 65 and baby boomers aged 45-64 | 756 | 455 (60.2%) | n/a | Standard camera | Private home |
| Berridge & Wetle (2020) | United States | To systematically compare mother–adult child dyads’ assessments of values at play in three forms of passive remote monitoring: location tracking, in-home activity sensors, and 24-hr Web cameras | Qualitative | Structured interviews | Older adults aged 61-95 and family members aged 28-66 | 18 | 18 (100%) | 77 | Standard camera | Private home |
| Bian et al. (2021) | Norway | To understand older adults’ perceptions and preferences on technologies that can be potentially used to measure frailty criteria in home settings | Qualitative | Focus groups | Older adults aged 65-84 | 15 | 9 (60%) | 71.3 | Standard camera and depth camera | Private home |
| Boissy et al. (2007) | Canada | To examine older adults’ requirements for robots in home telecare | Qualitative | Focus groups | Community-living older adults with disabilities aged 68-92 and healthcare professionals | 6 | 5 (83.3%) | 78.7 | Social robot equipped with standard camera | Private home |
| Cesta et al. (2018) | Italy | To systematically investigate the expectations of older adults of AAL technologies devoted to support their independent living | Mixed-methods | Focus groups and questionnaires | Older adults (age range n/a); healthcare professionals | 10 (focus group participants); 44 (questionnaire respondents) | n/a for focus group participants; 33 (74.4%) for questionnaire respondents | n/a for focus group participants; 79.5 for questionnaire participants | Social robot equipped with standard camera | Private home |
| Choi et al. (2021) | USA | To examine older adults' perceptions of Internet-of-Things smart home devices as part of a real-world feasibility study and describe what factors affect adoption of these technologies. | Qualitative | Semi-structured interviews | Older adults aged 65 and above | 37 | 29 (78.4%) | 77.6 | Standard (web) camera | Private home |
| Cinini et al. (2021) | Italy | To evaluate the impact of new technologies on older adults with respect to user engagement, individual wellbeing, and automated assessment of motor and cognitive functions. | Qualitative | Interviews | First round of interviews: Older adults aged 65-81; Second round of interviews: Older adults aged 65-87 | First round of interviews: 100; Second round of interviews: 202 | First round of interviews: (36) 36%; Second round of interviews: 119 (59%) | First round of interviews: 70; Second round of interviews: 74 | Standard camera | Private home |
| Claes et al. (2015) | Belgium | To explore attitudes and perceptions of adults of 60 years and older towards contactless monitoring of the activities of daily living | Quantitative | Questionnaires | Older adults aged 60-90 | 245 | 162 (67.8%) | 72.4 | Standard camera | Private home |
| Courtney et al. (2008) | United States | To investigate the factors that influence the willingness of older adults living in independent and assisted living continuing care retirement communities to adopt smart home technology | Qualitative | Semi-structured interviews and focus groups | Older adults aged 65 and above | 14 | n/a | n/a | Camera with identity-redacting privacy filter (e.g., silhouette extraction) | Private home in continuing care retirement community |
| Demiris (2009) | United States | To assess older adults’ and their caregivers’/ family members’ perceptions of the two sensing approaches and their privacy considerations associated with specific smart home examples | Qualitative | Semi-structured interviews | Older adults and their informal caregivers (e.g., family members, friends, spouses) | 20 | n/a | 74.5 | Standard camera | Private home in independent retirement community |
| Demiris et al. (2004) | United States | To assess the perceptions and expectations of seniors in regard to technology installed and operated in their homes with the purpose of improving their quality of life and/or monitoring their health status | Qualitative | Focus groups | Older adults aged 65 and above | 15 | 8 (53.3%) | n/a | Standard camera | Private home in continuing care retirement community |
| Demiris et al. (2008) | United States | To examine older adults’ attitudes toward specific sensor technologies and capture the level of willingness to allow installation of such technologies and to share associated personal data with other stakeholders | Qualitative | Focus groups | Older adults aged 65 and above | 14 | 9 (64.3%) | n/a | Camera with identity-redacting privacy filter (i.e., silhouette extraction) | Private home in continuing care retirement community |
| Demiris et al. (2009) | United States | To explore older adults’ privacy considerations for vision based recognition methods of technology applications in eldercare | Qualitative | Semi-structured interviews | Older adults aged 65 and above | 10 | 8 (80%) | n/a | Camera with identity-redacting privacy filter (i.e., silhouette extraction) | Private home |
| Dermody et al. (2021) | Australia | To explore factors that influence community-dwelling older adults’ readiness to adopt smart home technology to help guide the practical development, deployment and utilisation of smart home technology to meet the older person's needs | Qualitative | Semi-structured interviews and Focus groups | Older adults aged 68-87 | 19 | 14 (73.7%) | 78.9 | Standard camera | Private home |
| Elers et al. (2018) | New Zealand | To investigate how technologies that connect older adults to their informal and formal support networks could assist aging in place and enhance older adults’ health and well-being | Qualitative | Semi-structured interviews | Older adults aged 74-92 and their informal support network (e.g., family members, friends, neighbours) | 10 | 8 (80%) | n/a | Standard camera | Private home |
| Galambos et al. (2017) | United States | To explore the perceptions and preferences of older adults and their family members about a fall risk assessment system | Qualitative | Structured interviews | Older adults aged 67-98 | 13 | 8 (61.5%) | 86.9 | Standard camera and Microsoft Kinect (comprising standard camera and depth camera) | Private home |
| Gasteiger et al. (2022) | New Zealand | To explore the feasibility of using a dailycare service robot in the homes of community-dwelling older adults. To explore the usefulness of the robot and participants’ perceptions and experience of using it | Qualitative | Semi-structured interviews | Older adults aged 72-83 | 6 | 4 (66.7%) | 78 | Social robot equipped with depth camera | Private home |
| Ghorayeb et al. (2021) | United Kingdom | To understand older people’s views of smart homes and how their experience can influence these | Qualitative | Focus groups | Older adults aged 65-89 | 13 | 8 (61.5%) | n/a | “Silhouette sensor” – i.e., camera that records black-and-white silhouettes of users | Private home |
| Gibson et al. (2015) | Scotland | To explore how people with dementia and their family carers use assistive technology in their everyday lives, identify the types and range of devices they use, and the issues which influenced technology adoption within their usual care routines | Qualitative | Semi-structured interviews | Older adults with dementia aged 49-91; Carers of older adults with dementia aged 49-82 | 7 | n/a | 72 | Closed-circuit television camera | Private home |
| Gövercin et al. (2010) | Germany | To gather potential users’ opinions on ICT-based visual and wearable fall prediction and fall prevention systems for home use | Qualitative | Focus groups and questionnaires | Older adults aged 60-84 (categorised into high fall risk and low fall risk groups) and their relatives aged 50-85 | 16 | 12 (75%) | Older adults with high fall risk: 75;  Older adults with low fall risk: 68 | Standard camera, closed-circuit television camera and omnidirectional camera | Private home |
| Grace et al. (2017) | Canada | To understand the perceptions of seniors with heart failure regarding a smart-home system to autonomously monitor their physiological parameters, namely heart rate, blood pressure, temperature, weight, and respiration | Qualitative | Semi-structured interviews | Older adults aged 65 and above who are heart failure patients | 26 | 7 (26.9%) | 75.0 | Thermal camera | Private home |
| Harrington et al. (2021) | United States | To examine initial concerns related to the use and acceptance of socially assistive robots and identify potential determinants of robot acceptance in a sample of healthy community-dwelling older adults in the United States | Quantitative | Questionnaires | Older adults aged 60-92 | 44 | 31 (70.5%) | 74.3 | Social robot equipped with standard camera | Private home |
| Hattink et al. (2016) | The Netherlands | To integrate three previously developed assistive technology systems into one modular, multifunctional system, which can support people with dementia and carers throughout the course of dementia | Pre-test post-test control group design | Interviews and questionnaires | Older adults aged 62-96 | 42 | 21 (50%) | 80.6 in experimental group;  78.2 in control group | Standard camera | Private home |
| Igarashi & Nihei (2023) | Japan | To discover the preferences of older adults when they used sensors in their households | Quantitative | Questionnaires | Older adults aged 65 and above | 400 | 200 (50%) | Not reported | Standard camera | Private home |
| Jaschinski & Allouch (2015) | The Netherlands | To understand how prospective users perceive AAL technologies and to develop design guidelines in order to aid their acceptance | Qualitative | Focus groups and semi-structured interviews | Older adults aged 55-86 | 28 | 16 (57.1%) | 71.4 | Standard camera | Private home |
| Khosla et al. (2021) | Australia | To study the engagement and robot experience of older people with dementia while interacting with a social robot named Betty in the context of home-based care | Mixed-methods | Interviews and questionnaires | Older adults aged 75-85 | n/a | n/a | n/a | Social robot equipped with standard camera | Private home |
| Kim (2020) | United States | To advance knowledge of how sensor technology (e.g., Microsoft Kinect) should be implemented in the home of those with visual disabilities | Qualitative | Field observations and interviews | Community-dwelling older adults aged and above, with visual acuity levels worse than 20/70 | 20 | 16 (80%) | 72.9 | Microsoft Kinect (comprising standard camera and depth camera) | Private home |
| Kirchbuchner et al. (2015) | Germany | To shed light on the older adults’ expectations and fears related to innovations of ambient intelligence | Quantitative | Questionnaires | Older adults aged 48-84 | 60 | 42 (70%) | 67.7 | Standard camera | Private home |
| Kodate et al. (2021) | Ireland | To explore attitudes and perceptions of potential users regarding home-care robots which can provide companionship and support with activities of daily living | Quantitative | Questionnaires | Older adults aged 65 and above | 114 | n/a | n/a | Social robot equipped with standard camera | Private home |
| Krafft & Coskun (2009) | Sweden | To investigate how elderly perceive health smart homes, where results will be used to inform smart home design implications | Qualitative | Semi-structured interviews | Community-dwelling older adults aged 70-85 | 5 | n/a | n/a | Standard camera | Private home |
| Lapierre et al. (2018) | Canada | To explore the perception of older women at risk of falls regarding the use of a programmable video monitoring system | Qualitative | Semi-structured interviews | Older adults aged 65 and above | 6 | 6 (100%) | 73.7 | Camera with identity-redacting privacy filter (e.g., blurring) | Private home |
| Lukas et al. (2021) | Germany | To evaluate the security and acceptance of an intelligent home emergency call system used by older people receiving home care | Quantitative | Questionnaires | Adults aged 42-95 | 39 | 25 (64.1%) | 76.0 | Depth camera | Private home |
| Maan & Gunawardana (2017) | Australia | To investigate the barriers and perceptions in the use of AAL technologies amongst older Australians | Mixed-methods | Focus groups and questionnaires | Older adults aged 67-89 | 25 | 17 (68%) | n/a | Standard camera | Private home in retirement community |
| Mihailidis et al. (2008) | Canada | To gain insight into the acceptability of home monitoring technologies among older adults and baby boomers | Mixed-methods | Interviews and questionnaires | Older adults aged 65 and above;  Baby boomers aged 40-59 | 15 | 8 (53.3%) | n/a | Standard camera | Private home |
| Offerman-van Heek et al. (2019) | Germany | To investigate if different levels of necessity for care affects the perception of benefits and barriers of assistive technologies, the overall acceptance of AAL technologies, and the decisions for or against specific technologies in different situations | Quantitative | Questionnaires | Individuals aged 15-88;  Subgroups:  -Young (≤ 30 years)  -Middle (31-49 years)  -Best-agers (50-65 years)  -Seniors (> 65 years) | 24 | n/a | n/a | Standard camera | Private home |
| Steele et al. (2006) | Australia | To examine perspectives on the concept of sensing-based interaction and the challenges in designing sensor networks that are acceptable to the elderly | Qualitative | Focus groups | Older adults aged 65 and above | 13 | 7 (53.8%) | n/a | Standard camera | Private home |
| Rawtaer et al. (2021) | Singapore | To evaluate the feasibility of using commercially available sensors in a smart home to perform functional assessments of older adults to evaluate their ability to perform instrumental activities of daily living | Mixed-methods | Questionnaires | Older adults aged 65 and above | 35 | 20 (57.1%) | 71.7 | Standard (web) camera | Private home |
| Robinson et al. (2020) | United States | To explore how health information generated from in-home sensor technology can be tailored for in-home use by older adults and family members who may provide caregiving support | Mixed-methods | Focus groups and questionnaires | Older adults aged 61-97 and their family members aged 59-69 | 23 | 20 (87%) | 80 | Depth camera | Private home in independent living facility |
| Sánchez et al. (2019) | Norway | To explore older people’s attitudes to welfare technology | Qualitative | Semi-structured interviews | Older adults aged 79-91 | 9 | 5 (55.6%) | 83.1 | Standard camera | Private home |
| Sarkisian et al. (2003) | United States | To examine older adults’ concerns surrounding matters of acceptance, comfort, and perceived usefulness of a technology-rich home environment | Qualitative | Structured interviews | Older adults aged 65-75 | 17 | 10 (58.8%) | n/a | Standard camera | “Aware Home” living lab that simulates a private home environment |
| Sun & Yang (2021) | Norway | To develop and validate personal privacy predictive models based on patient’s preferences | Qualitative | Focus groups | Older adults aged 60 and above | 8 | n/a | n/a | Standard camera | Private home |
| van Hoof et al. (2011) | The Netherlands | To investigate the needs and motives, related to ageing-in-place, of the respondents receiving ambient intelligence technologies, and to investigate whether, and how, these technologies contributed to aspects of ageing-in-place | Qualitative | Interviews | Older adults aged 65-87 | 18 | 14 (77.8%) | 79.2 | Standard camera | Private home |
| Vaziri et al. (2017) | United States | To provide a better understanding of the effectiveness of ICT-based fall prevention for different subgroups and the indicators that determine the use of such technologies by older adults | Mixed-methods | Semi-structured interviews, field observations, workshops, focus groups, and questionnaires | Community-dwelling older adults aged 65 and above | 153 | 93 (60.8%) | Intervention group: 74.7;  Control group: 74.7 | Microsoft Kinect (comprising standard camera and depth camera) | Private home |
| Ziefle et al. (2011a) | Germany | To explore the acceptance of home-integrated ICT (hands-free equipment, camera, positioning system) | Mixed-methods | Focus groups and questionnaires | Individuals aged 24-73, with older adult subgroup | 74 | 39 (52.7%) | 68, 63, 58, 60 across four focus groups;  75 in questionnaire study | Standard camera | Private home |
| Ziefle et al. (2011b) | Germany | To explore attitudes towards the usage of video-based systems for long-term care of elderly or disabled people in smart home environments | Quantitative | Questionnaires | Individuals aged 17-95, with older adult subgroup | 46 | 25 (54%) | 72.5 | Standard camera | Private home |

*Note.* AAL = Active and Assisted Living; ICT = Information and Communications Technology.

Supplementary Table 4: Types of camera-based AAL technologies evaluated in the included studies.

| Study | Types of camera-based AAL technology under evaluation | | | | | | |
| --- | --- | --- | --- | --- | --- | --- | --- |
|  | Standard camera | Camera-equipped social robot | Camera with use of identity-redacting privacy filter | Microsoft Kinect (comprising standard and depth cameras) | Depth camera | Thermal camera | Other |
| Albina & Hernandez (2018) | x |  |  |  |  |  |  |
| Alkhatib et al. (2021) |  |  |  |  |  |  | Type of camera was not described – participants were free to conjure images of “aged care monitoring devices”, including (but not limited to) camera-based AAL technologies |
| Alsulami et al. (2016) | x |  |  |  |  |  |  |
| Arthanat et al. (2019) | x |  |  |  |  |  |  |
| Arthanat et al. (2020) | x |  |  |  |  |  |  |
| Beach et al. (2009) | x |  |  |  |  |  |  |
| Berridge & Wetle (2020) | x |  |  |  |  |  |  |
| Bian et al. (2021) | x |  |  |  | x |  |  |
| Boissy et al. (2007) |  | x |  |  |  |  |  |
| Cesta et al. (2018) |  | x |  |  |  |  |  |
| Choi et al. (2021) | x |  |  |  |  |  |  |
| Cinini et al. (2021) | x |  |  |  |  |  |  |
| Claes et al. (2015) | x |  |  |  |  |  |  |
| Courtney et al. (2008) |  |  | x |  |  |  |  |
| Demiris (2009) | x |  |  |  |  |  |  |
| Demiris et al. (2004) | x |  |  |  |  |  |  |
| Demiris et al. (2008) |  |  | x |  |  |  |  |
| Demiris et al. (2009) |  |  | x |  |  |  |  |
| Dermody et al. (2021) | x |  |  |  |  |  |  |
| Elers et al. (2018) | x |  |  |  |  |  |  |
| Galambos et al. (2017) | x |  |  | x |  |  |  |
| Gasteiger et al. (2022) |  | x |  |  |  |  |  |
| Ghorayeb et al. (2021) |  |  | x |  |  |  |  |
| Gibson et al. (2015) | x |  |  |  |  |  |  |
| Gövercin et al. (2010) | x |  |  |  |  |  |  |
| Grace et al. (2017) |  |  |  |  |  | x |  |
| Harrington et al. (2021) |  | x |  |  |  |  |  |
| Hattink et al. (2016) | x |  |  |  |  |  |  |
| Igarashi & Nihei (2023) | x |  |  |  |  |  |  |
| Jaschinski & Allouch (2015) | x |  |  |  |  |  |  |
| Khosla et al. (2021) |  | x |  |  |  |  |  |
| Kim (2020) |  |  |  | x |  |  |  |
| Kirchbuchner et al. (2015) | x |  |  |  |  |  |  |
| Kodate et al. (2021) |  | x |  |  |  |  |  |
| Krafft & Coskun (2009) | x |  |  |  |  |  |  |
| Lapierre et al. (2018) |  |  | x |  |  |  |  |
| Lukas et al. (2021) |  |  |  |  | x |  |  |
| Maan & Gunawardana (2017) | x |  |  |  |  |  |  |
| Mihailidis et al. (2008) | x |  |  |  |  |  |  |
| Offerman-van Heek et al. (2019) | x |  |  |  |  |  |  |
| Steele et al. (2006) | x |  |  |  |  |  |  |
| Rawtaer et al. (2021) | x |  |  |  |  |  |  |
| Robinson et al. (2020) |  |  |  |  | x |  |  |
| Sánchez et al. (2019) | x |  |  |  |  |  |  |
| Sarkisian et al. (2003) | x |  |  |  |  |  |  |
| Sun & Yang (2021) | x |  |  |  |  |  |  |
| van Hoof et al. (2011) | x |  |  |  |  |  |  |
| Vaziri et al. (2017) |  |  |  | x |  |  |  |
| Ziefle et al. (2011a) | x |  |  |  |  |  |  |
| Ziefle et al. (2011b) | x |  |  |  |  |  |  |
